# Genome-Wide Discovery of Microsatellite Markers from Diploid Progenitor Species, *Arachis duranensis* and *A. ipaensis*, and Their Application in Cultivated Peanut (*A. hypogaea*)

**DOI:** 10.3389/fpls.2017.01209

**Published:** 2017-07-18

**Authors:** Chuanzhi Zhao, Jingjing Qiu, Gaurav Agarwal, Jiangshan Wang, Xuezhen Ren, Han Xia, Baozhu Guo, Changle Ma, Shubo Wan, David J. Bertioli, Rajeev K. Varshney, Manish K. Pandey, Xingjun Wang

**Affiliations:** ^1^Biotechnology Research Center, Shandong Academy of Agricultural Sciences and Shandong Provincial Key Laboratory of Crop Genetic Improvement, Ecology and Physiology Jinan, China; ^2^College of Life Sciences, Shandong Normal University Jinan, China; ^3^United States Department of Agriculture – Agricultural Research Service, Crop Protection and Management Research Unit, Tifton GA, United States; ^4^International Crops Research Institute for the Semi-Arid Tropics (ICRISAT) Hyderabad, India; ^5^Center for Applied Genetic Technologies, University of Georgia, Athens GA, United States

**Keywords:** wild peanut, genome sequence, microsatellites, molecular markers, quantitative trait locus (QTL)

## Abstract

Despite several efforts in the last decade toward development of simple sequence repeat (SSR) markers in peanut, there is still a need for more markers for conducting different genetic and breeding studies. With the effort of the International Peanut Genome Initiative, the availability of reference genome for both the diploid progenitors of cultivated peanut allowed us to identify 135,529 and 199,957 SSRs from the A (*Arachis duranensis*) and B genomes (*Arachis ipaensis*), respectively. Genome sequence analysis showed uneven distribution of the SSR motifs across genomes with variation in parameters such as SSR type, repeat number, and SSR length. Using the flanking sequences of identified SSRs, primers were designed for 51,354 and 60,893 SSRs with densities of 49 and 45 SSRs per Mb in *A. duranensis* and *A. ipaensis*, respectively. *In silico* PCR analysis of these SSR markers showed high transferability between wild and cultivated *Arachis* species. Two physical maps were developed for the A genome and the B genome using these SSR markers, and two reported disease resistance quantitative trait loci (QTLs), *qF2TSWV5* for tomato spotted wilt virus (TSWV) and *qF2LS6* for leaf spot (LS), were mapped in the 8.135 Mb region of chromosome A04 of *A. duranensis*. From this genomic region, 719 novel SSR markers were developed, which provide the possibility for fine mapping of these QTLs. In addition, this region also harbors 652 genes and 49 of these are defense related genes, including two NB-ARC genes, three LRR receptor-like genes and three WRKY transcription factors. These disease resistance related genes could contribute to resistance to viral (such as TSWV) and fungal (such as LS) diseases in peanut. In summary, this study not only provides a large number of molecular markers for potential use in peanut genetic map development and QTL mapping but also for map-based gene cloning and molecular breeding.

## Introduction

Cultivated peanut or groundnut (*Arachis hypogaea* L.), is a source of high quality edible oil and protein, and is one of the most important oil crops worldwide. Peanut is widely planted in China, India and the United States. The world’s total consumption of peanut is about 29 million metric tons per year^1^, contributing to an estimated production value of about $35 billion (Guo et al., 2011). However, peanut is often grown on marginal soils with lesser inputs and usually intercropped with cereals in many countries. Peanut production and productivity is often constrained by several biotic and abiotic factors, such as drought, salinity, bacterial wilt disease, and leaf spot disease (Krishna et al., 2015). Cultivated peanut is an allotetraploid (AABB, 2n = 4 × = 40) derived from hybridization between *A. duranensis* (AA, 2n = 2 × = 20) and *A. ipaensis* (BB, 2n = 2 × = 20) (Lavia et al., 2011). During the past decade, significant progress has been made in developing genomic resources which facilitated several successful efforts of trait mapping and molecular breeding in peanut (Pandey et al., 2012, 2016; Varshney et al., 2013). These studies used low density genetic maps and hence could not provide the optimum level of resolution for trait dissection and discovery of candidate genes. Nevertheless, some of these studies produced very good results despite deploying the minimal genomic and genetic resources including SSRs available at that time (Varshney et al., 2013).

Microsatellites, or simple sequence repeats (SSRs), are DNA fragments consisting of tandemly repeated short units (1–6 bp) that are present in both protein coding and non-coding regions of the genome (Gupta et al., 1996; Squirrell et al., 2003; Haq et al., 2014). SSRs have become a common tool and were broadly used in plant genetics analysis and breeding programs, owing to their characteristics of simplicity, abundance, ubiquity, variation, co-dominance, and multi-allelism among genomes (Powell et al., 1996). The polymorphism of SSRs generated from the number of repeat units can easily be detected by PCR method using primers designed according to the flanking sequences. Although the development of SSR markers was previously expensive, labor intensive and time consuming (Varshney et al., 2002), the availability of low-cost sequencing data using next-generation sequencing (NGS) made this task faster and less expensive in identifying genome-wide structural variations including SSRs as potential genetic markers in many crops (Pandey et al., 2016).

Simple sequence repeats derived from expressed sequence tags, transcriptome libraries, and genomic libraries are referred to as EST-SSRs, transcriptome-SSRs and g-SSRs, respectively. EST-SSRs and transcriptome-SSRs are derived from the CDS region of genes with high selection pressure, leading to low polymorphism in contrast to SSRs from non-CDS regions of the genome (Cho et al., 2000; Shore et al., 2010). In bread wheat, it was shown that the polymorphism rate in EST-SSRs is lower than that in g-SSRs, suggesting that g-SSRs can serve as valuable complements to EST-SSRs and transcriptome-SSRs (Balfourier et al., 2007; Han et al., 2015). A number of studies reported development of SSR markers in peanut, for example, EST-SSRs derived from cDNA libraries (Liang et al., 2009; Song et al., 2010), SSRs from bacterial artificial chromosome (BAC)-end sequences (Wang et al., 2012), and transcriptome-SSRs from a transcriptome library of developing seeds (Zhang et al., 2012; Huang et al., 2016; Zhong et al., 2016; Zhou et al., 2016). Such studies were very limited and hence the available SSRs are insufficient for conducting moderate to high resolution genetic studies in peanut.

The peanut genomics research witnessed significant milestone in 2016 by completing genome sequencing of the diploid progenitors of cultivated peanut, i.e., *A. duranensis* and *A. ipaensis* (Bertioli et al., 2016; Chen et al., 2016). Although one of these sequencing efforts developed genome-wide SSRs for *A. duranensis* (Chen et al., 2016), the application of these SSRs is limited due to lack of pseudomolecule-level information. The high quality genome assemblies developed by the International Peanut Genome Initiative (IPGI) and the Peanut Genome Consortium (PGC) provided opportunity for developing genetic markers from genome-wide structural variations in peanut (Bertioli et al., 2016). As members of IPGI and the joint effort to accelerate marker-assisted selection (MAS) in peanut, we identified and developed g-SSRs from *A. duranensis* and *A. ipaensis*. The frequency and distribution of motif length, type, and repeat number of SSRs in the genomes of these two *Arachis* species were also compared. Using these SSRs, we constructed a high-density SSR physical map of wild peanuts. The positions of these SSRs were compared with the previously developed *Arachis* SSR markers. To evaluate the application value of these SSRs, we studied their polymorphism in different species. This study found these newly developed g-SSR markers very useful and could facilitate the advancement of many basic and applied genomic studies in peanut, including fine mapping of quantitative trait loci (QTLs) for important agronomic traits, positional-based gene cloning, molecular breeding, and diversifying the cultivated genepool using wild relatives of peanut.

## Materials and Methods

### Plant Material and DNA Extraction

Two cultivated peanut genotypes namely Tifrunner and Fenghua 1, two diploid ancestors namely *A. duranensis* and *A. ipaensis*, a synthetic amphidiploid namely IpaDur (*A. ipaensis* × *A. duranensis*), and a (Fenghua1 × IpaDur) F_1_ individual were used in this study for SSR identification and validation. Genomic DNA of *A. duranensis, A. ipaensis, Tifrunner* and (Fenghua1 × IpaDur) F_1_ was isolated from seeds using Plant Genomic DNA Extraction Kit (TIANGEN, Beijing, China) according to the instructions of the manufacturer^2^.

### Validation of SSRs by PCR Amplification

Polymerase chain reactions (PCRs) were performed in a total of volume of 20 μl that contained 0.2 mM dNTP Mix, 0.5 μM of each primers, 0.5 U of rTaq DNA polymerase (Takara, Dalian, China), 2.0 mM MgCl2, 1x PCR buffer and 70 ng of template DNA. The PCR program was as following: 94°C for 4 min, 35 cycles of 30 s at 94°C, 30 s at annealing temperatures (refer to Supplementary Tables S1, S2) and 30 s at 72°C, 7 min at 72°C. PCR products were separated via 6.5% denaturing polyacrylamide gels. Gels were fast silver stained and photographed.

### Identification of SSRs

The whole genome sequences of *A. duranensis* and *A. ipaensis* were downloaded from PeanutBase^3^. The genome sequences were used to identify SSR loci using Perl scripts software MISA^4^ with the default parameters. The identification criteria were: mono-nucleotide repeats motif with at least 12 repeats, di-nucleotide with six, tri- and quad-nucleotide with five, penta and hexa-nucleotide with four repeats. Compound microsatellites were defined as those with the interval between two repeats motifs shorter than 100 nt as previous reports (Wang et al., 2015; Deng et al., 2016; Liu et al., 2016).

### Designing Primers for SSRs

Primers were designed from flanking sequences of the identified SSRs using Primer 3 software^5^ with the following parameters: 18–27 bp primer length, 57–63°C melting temperature, 30–70% GC content and 100–300 bp product size. In order to run Primer 3.0, another two Perl scripts, p3_in.pl and p3_out.pl were used. The p3_in.pl and p3_out.pl were used to create a primer3 input file and to calculate and merge all information.

### *In Silico* Evaluation of Genome-Wide SSRs in Wild and Cultivated Peanut Species

The amplification efficiency of newly developed SSRs was evaluated using *in silico* PCR method. The genome sequences of *A. duranensis, A. ipaensis* and the scaffold sequences of Tifrunner (unpublished data) were used as templates. The software e-PCR (Version: 2.3.12^6^) was used for *in silico* PCR with the default parameters: 2 bp mismatch, 1 bp gap, 50 bp margin, and 50–1000 bp product size (Shi et al., 2014; Deng et al., 2016). The polymorphism of SSR primers were tested by comparing the repeat numbers of the particular SSR in different *Arachis* species.

### Integration of the Newly Developed SSRs with the Available SSR Map

The newly developed SSRs were physically mapped on the 10 pseudomolecules each of *A. duranensis* and *A. ipaensis* according to their genomic location (Bertioli et al., 2016). For the available published SSR markers of *Arachis* genetic linkage maps (Shirasawa et al., 2013), the original sequences of ESTs, GSS, BAC and transcriptome sequences were downloaded from GenBank^7^ for sequence alignment. To integrate the new SSRs with other publicly available SSR maps (Shirasawa et al., 2013), the original sequences of public SSRs were mapped against *Arachis* genome sequences using BLAST software^8^.

## Results

### Genome-Wide Discovery of SSRs in Peanut Diploid Progenitors

The available genome sequences of *A. duranensis* and *A. ipaensis* were searched for microsatellites with different types of desirable repeat motifs from mono- to hexa-nucleotide. A total of 135,529 and 199,957 SSRs were identified from the 1,084.3 and 1,353.8 Mb genomic sequences of *A. duranensis* and *A. ipaensis*, respectively. The overall frequency occurrence of SSRs was 125.0 and 147.7 SSRs per Mb, or one SSR every 8.0 and 6.7 Kb in *A. duranensis* and *A. ipaensis*, respectively (**Table 1**). The length, type and repeat numbers of SSRs had high correlations between *A. duranensis* and *A. ipaensis* genomes.

**Table 1:** Overall frequency of SSRs in *A. duranensis* and *A. ipaensis.*

Species	Microsatellites number	Genome length (bp)	Frequency	
			Per Mb	One every (kb)
*A. duranensis*	135,529	1,084,261,490	125.0	8.0
*A. ipaensis*	199,957	1,353,826,449	147.7	6.7

A total of 378 and 392 types of SSR motifs were detected in these two *Arachis* species, respectively. Among them, there were 2, 4, 10, 26, 89, 247 types, and 2, 4, 10, 25, 93, 258 types of mono- to hexanucleotide repeats in *A. duranensis* and *A. ipaensis*, respectively. The type and number of mono-, di-, and tri- SSR motifs were similar in frequency in these two progenitor species. A total of 25 types of tetra- SSR motifs were found in both *A. duranensis* and *A. ipaensis* genomes except AGCC/CGGT, which could be detected only in *A. duranensis.* In addition, 10 types of penta- SSR motifs (three in *A. duranensis*, seven in *A. ipaensis*) and 89 types of hexa- SSR motifs (39 in *A. duranensis*, 50 in *A. ipaensis*) were found in only one of these two progenitor species.

**FIGURE 1:**
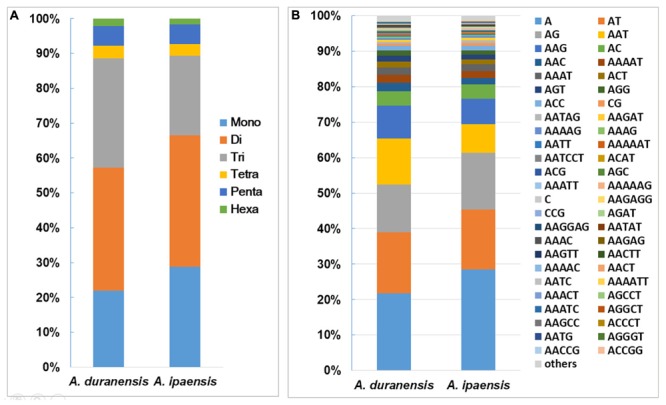
Distribution of SSR motifs in *Arachis* species. **(A)** Distribution of SSR motif length from mono- to hexanucleotide repeats in *A. duranensis* and *A. ipaensis*. Flexible relaxed criteria were used to identify SSR with minimum repeats number of 12, 6, 5, 5, 4, and 4 for mono- to hexanucleotide repeat motifs. **(B)** The vertical axis indicates the abundance (%) of SSRs with different type of motifs.

Among the 135,529 SSRs obtained from *A. duranensis*, the dinucleotide repeats were most abundant (47,805) with a proportion of 35.3%, followed by tri- (42,529, 31.4%), mono- (29689, 21.9%), penta- (7,658, 5.7%), tetra- (4,988, 3.7%), and hexa- (2,860, 2.1%) nucleotide SSRs. For *A. ipaensis*, dinucleotide (75,334, 37.7%) also represented the most abundant SSR motifs, followed by mono- (57589, 28.8%), tri- (45,717, 22.5%), penta- (11,092, 5.5%), tetra- (6,736, 3.4%), and hexa- (3,489, 1.7%) nucleotide SSRs (**Figure 1A**). These results indicated that the distributions of the motif length in the assembled genomic sequences of *A. duranensis* and *A. ipaensis* were almost identical, i.e., mono-, di-, tri- repeats represented the most abundant motifs and accounted for similar proportions, whereas tetra-, penta-, and hexanucleotide repeats were relatively uncommon.

**FIGURE 2:**
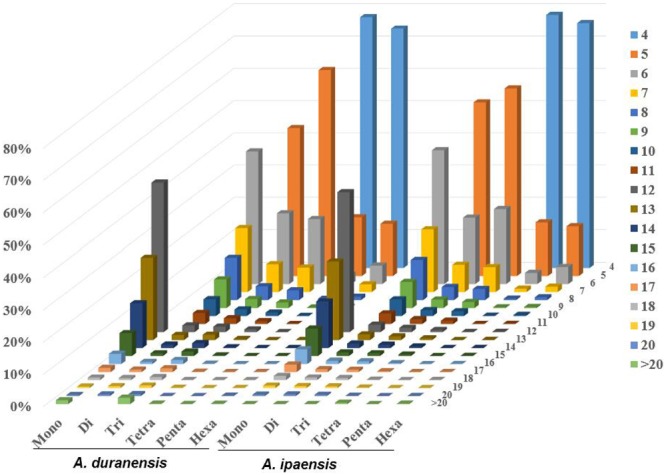
Distribution of SSR motif repeat number from mono- to hexanucleotide. The vertical axis shows the abundance of SSRs that have different motif repeat number (from 4 to >20), which are discriminated by legends of different colors.

The distributions of the top 50 SSR types in the assembled genomic sequences of *A. duranensis* and *A. ipaensis* are presented in **Figure 1B**. Among them, A/T had the highest occurrence (21.8% in *A. duranensis*, 28.4% in *A. ipaensis*), followed by AT/AG (17.3% in *A. duranensis*, 16.9% in *A. ipaensis*), AG/CT (13.4% in *A. duranensis*, 16.0% in *A. ipaensis*) and AAT/ATT (9.3% in *A. duranensis*, 7.3% in *A. ipaensis*) (**Figure 1B**). Specifically, the major motifs of mono- to hexanucleotide repeats were A, AT, AAT, AAAT, AAAAT and AAAAAT, respectively, in which AT motifs were rich. SSRs with CG-rich repeats were rare in both *Arachis* species. In addition, we observed that the abundances of SSRs decreased with the motif repeat number increasing, and the rate of this change was the fastest for hexa- and pentanucleotide repeats, followed by tetra-, tri-, di, and monoucleotide repeats (**Figure 2**).

**FIGURE 3:**
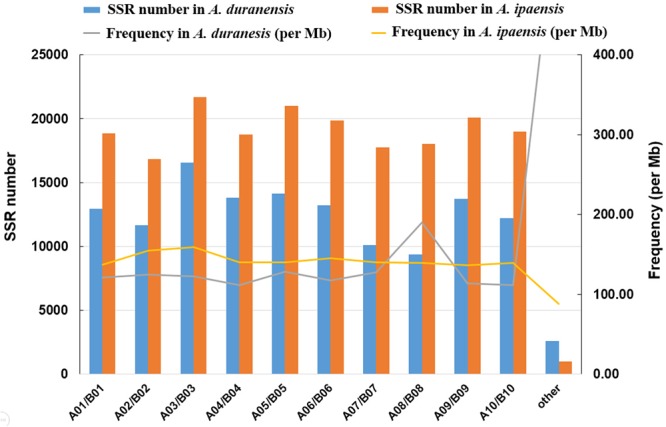
Chromosome-wide distribution of SSRs in *A. duranensis* and *A. ipaensis* genomes.

We analyzed the distribution of SSRs on each chromosome of *A. duranensis* (A genome) and *A. ipaensis* (B genome). Among the 10 chromosomes of *A. duranensis*, chromosome A03 (16,546) had the largest number of SSRs, followed by chromosomes A05, A04, A09, A06, A01, A10, A02, A07, and A08. The top five largest chromosomes of *A. duranensis* are A03, A04, A09, A06 and A05, therefore, chromosome length was not associated with the number of SSRs per chromosome. The differences in densities of SSRs on different chromosomes were significant, ranging from 111.63 SSRs/Mb to 189.58 SSRs/Mb with average of 124.69 SSRs/Mb. The density on chromosome A08 was the highest (189.58 SSRs/Mb), while that on chromosome A10 was the lowest (111.63 SSRs/Mb). For *A. ipaensis*, chromosome B03 (21,680) had the largest number of SSRs, followed by chromosomes B05, B09, B06, B10, B01, B04, B08, B07, and B02. Similarly, the numbers of SSRs were not in accordance with the length of each chromosome. Compared to *A. duranensis*, the differences in densities of SSRs on different chromosomes were not significant in *A. ipaensis*, ranging from 159.28 SSRs/Mb to 136.49 SSRs/Mb with average 142.43 SSRs/Mb. As mentioned above, the average density of SSRs on the entire genome of *A. ipaensis* was higher than that of *A. duranensis*. We noted that the number and densities of SSRs in each chromosome of *A. ipaensis* was higher than that in the counterpart chromosome of *A. duranensis*, except for A08 and B08 (**Figure 3**).

### Development of Primers for Newly Identified Genome-Wide SSRs

According to the flanking sequences of the identified SSRs from *A. duranensis* and *A. ipaensis*, amplification primers for 51,354 and 60,893 SSRs were successfully designed, accounting for 39.40 and 31.58% of 130,346 and 192,821 SSR loci, respectively (**Table 2**). We failed to generate specific amplification primers for the remaining SSRs mostly due to the limited length of flanking sequences from each side of the SSRs, especially for mononucleotide type SSRs. The densities of the SSR markers that could be amplified were 49.13 per Mb (or one every 20.36 kb) and 44.98 per Mb (or one every 22.23 kb) in the *A. duranensis* and *A. ipaensis* genomes, respectively (**Table 3**). Among these SSR markers, dinucleotide repeats SSRs were most abundant (23595, 30304) with proportions of 45.95% and 49.60% in *A. duranensis* and *A. ipaensis*, respectively. Trinucleotide repeats were the second most popular SSRs in both diploid progenitor species, accounting for more than one-third of total SSRs, followed by hexa-, penta-, and tetranucleotide SSRs.

**Table 2:** Summary of SSR markers in *A. duranensis* and *A. ipaensis.*

Species	*A. duranensis*				*A. ipaensis*			
Repeats	Number	Types	Proportion (%)	Frequency (per Mb)	Number	Types	Proportion (%)	Frequency (per Mb)
Mono-	307	4	0.60	0.29	513	4	0.84	0.38
Di-	23,595	12	45.95	22.57	30,304	12	49.60	22.38
Tri-	20,227	60	39.39	19.35	21,667	60	35.47	16.00
Tetra-	2,775	117	5.40	2.65	3,174	120	5.20	2.34
Penta-	3,183	387	6.20	3.04	3,928	417	6.43	2.90
Hexa-	1,267	674	2.47	1.21	1,505	747	2.46	1.11
Total	51,354	1,254	100	49.13	61,091	1,360	100	45.12

**Table 3:** Distribution of SSR markers in chromosomes of *A. duranensis* and *A. ipaensis.*

Species	*A. duranensis*				*A. ipaensis*			
Chromosome	Chromosome size (Mb)	Number	Frequency (per Mb)	Frequency (one every kb)	Chromosome size (Mb)	Number	Frequency (per Mb)	Frequency (one every kb)
1	107.04	5,305	49.56	20.18	137.41	5,746	41.82	23.91
2	93.87	4,610	49.11	20.36	109.00	5,548	50.90	19.65
3	135.06	6,734	49.86	20.06	136.11	7,343	53.95	18.54
4	123.56	5,267	42.63	23.46	133.62	5,898	44.14	22.65
5	110.04	5,548	50.42	19.83	149.90	6,674	44.52	22.46
6	112.75	5,200	46.12	21.68	137.15	6,131	44.71	22.37
7	79.13	4,056	51.26	19.51	126.35	5,628	44.54	22.45
8	49.46	4,089	82.67	12.10	129.61	5,660	43.67	22.90
9	120.67	5,192	43.03	23.24	147.09	6,382	43.39	23.05
10	109.46	4,773	43.60	22.93	136.18	5,960	43.77	22.84
Others	4.32	580	134.17	7.45	11.42	121	10.60	94.36
Total	1,045.36	51,354	49.13	20.36	1,353.83	61,091	44.98	22.23

The motif types and the distributions of developed SSR markers were consistent with that of SSR loci in *A. duranensis* and *A. ipaensis* genomes, in which AT-rich repeat motifs were abundant, while the CG-rich repeat patterns were rare (Supplementary Tables S1, S2). For example, AT/TA (11,829, 16,227) repeats were the most abundant SSRs, contributing to 50.13% and 53.55% of total dinucleotide SSR markers in *A. duranensis* and *A. ipaensis*, respectively. Moreover, we analyzed the distribution of SSR markers on different chromosomes of *A. duranensis* and *A. ipaensis* (**Table 3**). For *A. duranensis*, chromosome A03 had the largest number (6,734) of SSR markers, followed by chromosomes A05 and A01, containing 5,548 and 5,305 SSR markers, respectively. In *A. ipaensis*, chromosome B03 (7,343) had more SSR markers than other chromosomes (**Table 3**). Chromosome A07 (4,056) and B02 (5,548) had the fewest SSR markers in *A. duranensis* and *A. ipaensis*, respectively (**Table 3**). Chromosome A08 had a density of 82.67 SSR markers per Mb, which was considerably higher than that of other chromosomes. The densities of SSRs on other chromosomes were not different and ranged from 41.82 (B01) to 53.59 per Mb (B03) (**Table 3**).

### Initial Validation through *In Silico* PCR Analysis

To analyze the amplification efficiency and specificity of the developed SSR markers, all SSR markers were subjected to *in silico* PCR analysis based on the genome sequences of *A. duranensis, A. ipaensis* and the scaffold sequences of Tifrunner (unpublished data), a tetraploid *A. hypogaea* variety which was selected for whole genome sequencing. The numbers of *in silico* PCR product(s) were recorded and summarized (**Table 4**). The *in silico* PCR results demonstrated that more than three-quarters of the SSR markers (39,922 out of 51,354) from AA genome only generated one specific PCR product in AA genome. And 4,617 (8.99%), 1,562 (3.04%), and 5,253 (10.23%) SSR markers from AA genome generated 2, 3, and >3 PCR products, respectively, in *A. duranensis* genome sequences. A total of 15,734 (30.64%), 2,187 (4.26%), 969 (1.89%), and 4,091 (7.97%) SSR markers from *A. duranensis* generated 1, 2, 3, and >3 PCR products, respectively, in *A. ipaensis* genome. More than half of the developed SSR markers from the AA genome could not generate PCR product(s) in the BB genome (**Table 4**).

**Table 4:** Generated number of *in silico* PCR products by genome-wide SSR markers in *A. duranensis, A. ipaensis*, and *A. hypogaea* (Tifrunner) genomes.

Genomes	*In silico* PCR in	Zero	One	Two	Three	>Three	Total
*A. duranensis*	*A. duranensis*	0 (0%)	39,922 (77.74%)	4,617 (8.99%)	1,562 (3.04%)	5,253 (10.23%)	51,354 (100.00%)
	*A. ipaensis*	28,373 (55.25%)	15,734 (30.64%)	2,187 (4.26%)	969 (1.89%)	4,091 (7.97%)	
	*A. hypogaea* (Tifrunner)	14,541 (28.32%)	18,793 (36.60%)	9,514 (18.53%)	1,844 (3.59%)	6,662 (12.97%)	
*A. ipaensis*	*A. duranensis*	53,896 (88.22%)	6,762 (11.07%)	345 (0.56%)	45 (0.07%)	43 (0.07%)	61,091 (100.00%)
	*A. ipaensis*	91 (0.15%)	43,954 (71.95%)	5,402 (8.84%)	2,379 (3.89%)	9,365 (1.33%)	
	*A. hypogaea* (Tifrunner)	12,019 (19.67%)	25,194 (41.24%)	11,254 (18.42%)	2,682 (4.39%)	9,942 (16.27%)	

For SSR markers from *A. ipaensis*, 91 (0.15%), 43,954 (71.95%), 5,402 (8.84%), 2,379 (3.89%), and 9,365 (1.33%) of them generated 0, 1, 2, 3, and >3 PCR products, respectively, in *A. ipaensis* genome. A total number of 53,896 (88.22%), 6,762 (11.07%), 345 (0.56%), 45 (0.07%), and 43 (0.07%) of them generated 0, 1, 2, 3, and >3 PCR products, respectively, from *A. duranensis* genome (**Table 4**).

Although all of these SSR markers were derived from the wild diploid progenitor species, we found that a large portion of them could be amplified in cultivated peanut Tifrunner. A total of 36,813 (71.68%) SSR markers from *A. duranensis* and 49,072 (80.33%) from *A. ipaensis* could generate at least one PCR product. A total of 18,793 (36.60%) and 25,194 (41.24%) SSR markers derived from *A. duranensis* and *A. ipaensis*, respectively, generated only one specific PCR product in Tifrunner (**Table 4**). These results suggested that SSR markers from wild *Arachis* species had high transferability between wild and cultivated *Arachis* species, implying that these SSR markers have high application value in peanut molecular breeding.

**FIGURE 4:**
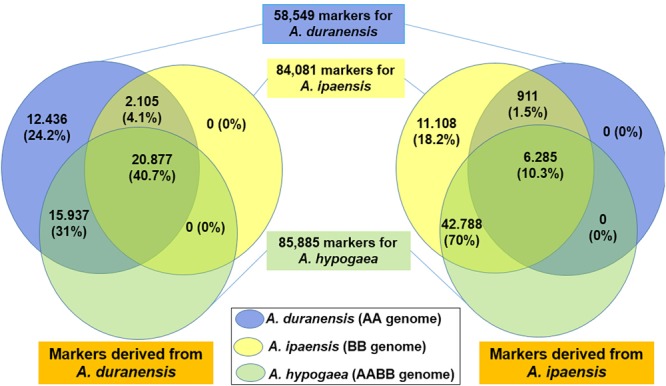
*In silico* PCR analysis of SSR markers in *A. duranensis, A. ipaensis*, and *A. hypogaea.*

In total, 58,549, 84,081 and 85,885 SSR markers could generate at least one PCR product in diploid progenitors (*A. duranensis, A. ipaensis*), and cultivated tetraploid (*A. hypogaea*), respectively (**Figure 4**). Among them, 27,161 markers could be used in *A. duranensis, A. ipaensis* and Tifrunner.

### Amplification and Polymorphism Analysis of SSR Markers in Different *Arachis* Species

**FIGURE 5:**
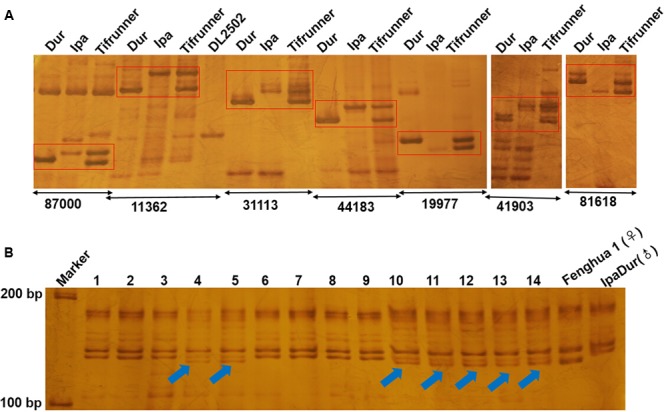
Electrophoresis analysis of genomic SSR in peanut. **(A)** Partial polymorphic SSR markers in *A. duranensis, A. ipaensis* and Tifrunner. **(B)** SSR detection using SSR marker Araip.B07_130994 in hybrid F_1_ of Fenghua1 x IpaDur. 1–14 represent harvest F_1_ offsprings, the arrows indicate the true hybrids.

To confirm amplification of the SSR markers, 113 of them were randomly selected for PCR amplification in *A. duranensis, A. ipaensis* and Tifrunner. Genomic DNA was used as the template. Our results showed that 111 (98.23%) of the 113 SSRs could produce clear amplification products. The results demonstrated that most of the SSRs displayed polymorphism in *A. duranensis* and *A. ipaensis*. Interestingly, many of these polymorphism sites could be observed in the tetraploid cultivated peanut (**Figure 5A**). These polymorphic markers can be used for distinguishing the alleles from the AA or BB ancestor species.

### Construction of SSR-Based Physical Map of *A. duranensis* and *A. ipaensis*

**FIGURE 6:**
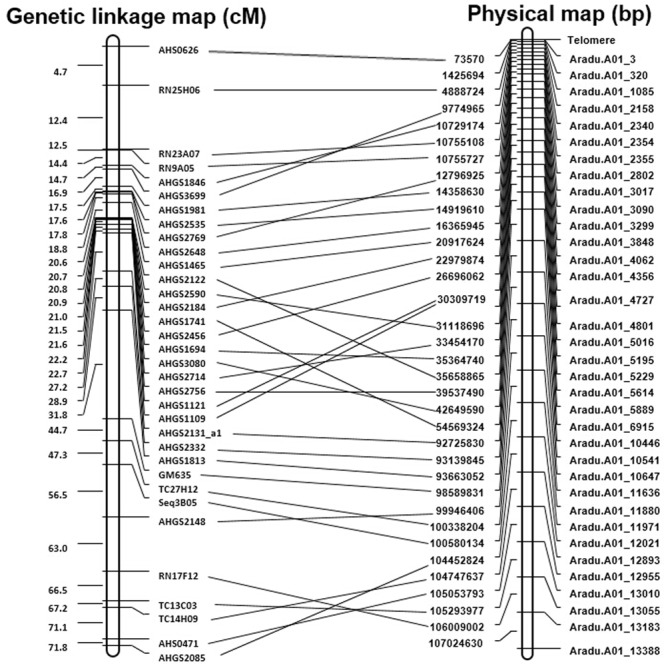
Comparison of the genetic linkage maps with physical map of A01 chromosome. This figure shows comparison on the genetic linkage map of Shirasawa et al. (2013) with the physical map.

All the developed SSR markers were anchored to the draft genome sequences of *A. duranensis* and *A. ipaensis*. As a result, 50,774 of 51,354 (98.9%) markers from *A. duranensis* and 60,970 of 61,091 (99.8%) markers from *A. ipaensis* were mapped to the 20 *Arachis* chromosomes, respectively (Supplementary Tables S1, S2). For each marker, the SSR repeat motifs, position in chromosomes, primer sequences, annealing temperature (Tm), and the length of PCR production, etc. listed in Supplementary Tables S1, S2. These maps provided a platform for marker assisted breeding, gene and QTL mapping in peanut. Recently, a reference integrated map was constructed using a comparative genomic method with 3,693 marker loci anchored to 20 consensus LGs of the peanut A and B sub-genomes (Shirasawa et al., 2013). The SSR-based physical maps were compared with the genetic linkage maps reported previously (Shirasawa et al., 2013). There were 84 marker loci in A01 linkage group, and most of them were SSRs developed from ESTs. A large portion (34 out of 84, 40.5%) of the EST-SSRs matched with the SSR markers from this study. The alignment of the physical map with the known linkage groups of peanut provided enough markers for increasing the density of genetic map for further fine mapping (**Figure 6**).

## Discussion

### Development of Large Number of Genome-Wide Novel SSR Markers: An Important Genomic Resource for Peanut Research Community

SSRs markers have been very useful for genetic analysis, mapping and breeding in several crop species. Although the current trend is more toward using single nucleotide polymorphism (SNP) markers in advanced research organizations in peanut (Pandey et al., 2017), the majority of research organizations with low to moderate genomics facilities still use SSRs in their research. In this context, a large number of genome-wide SSRs were previously only available for *A. duranensis* (Chen et al., 2016) and their limitation for tracking to a physical location in a specific genomic region restricts their use in genetic studies. Further, there was no effort to make available a large number of SSRs from *A. ipaensis*. In this context, we identified large scale genome-wide SSRs from both diploid progenitor species, i.e., 135,529 SSRs from the A genome (*A. duranensis*) and 199,957 SSRs from the B genome (*A. ipaensis*). In order to deploy these important structural variations in multiple genetic and breeding studies, we also developed primers for 112,247 genome-wide SSRs (51,354 SSRs for A genome and 60,893 SSRs for B genome). These SSRs will be a great resource for conducting genetic and genomic studies such as genetic diversity, genetic mapping, marker-trait association, and molecular breeding.

### Application of SSR-Based Physical Map in Fine Mapping Reported Peanut QTLs

**FIGURE 7:**
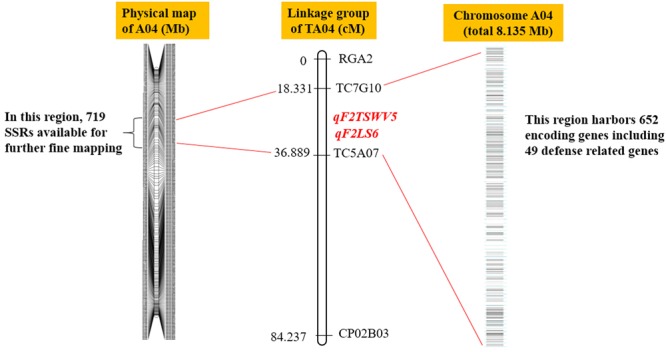
Comparison of two known QTLs in peanut physical map and genetic linkage map.

In the last decade, several efforts were employed for identification of QTLs linked to multiple phenotypic traits of peanut, which provides the basis for MAS. However, due to the lack of molecular markers, most of the peanut QTLs were far away from the target traits in genetic distance. Here, the physical map of *A. duranensis* and *A. ipaensis* allowed identification of molecular markers closer to the target traits. For example, previous studies reported a genomic region (TC5A07-TC7G10) in peanut linkage group TA04, which harbored a single QTL for tomato spotted wilt virus (TSWV)and named as *qF2TSWV5* (Wang et al., 2013). The QTL of *qF2TSWV5* account for 23.02% of TWSV phenotypic variance (PV) (Wang et al., 2013). In this region, four QTLs for leaf spot (LS) were also identified and named as *qF2LS6*, accounting for 10.08%–24.19% of phenotype variation (Wang et al., 2013). Markers TC5A07 and TC7G10 were derived from EST clones DQ099196 and DQ099144, respectively. According to the sequences of DQ099196 and DQ099144, TC5A07 and TC7G10 were successfully located in region of 8.135 Mb of chromosome 4 in *A. duranensis* (A04). Upon comparing this region in the SSR-based physical map of *A. duranensis*, we found a sequence of 8.135 Mb between the Aradu.A04_43370 and Aradu.A04_45137 genes (**Figure 7**). This region contained 719 newly developed genomic-SSR markers (**Figure 7** and Supplementary Table S3). These large number of SSRs provide the possibility for further fine mapping or even cloning of the QTLs or genes that confer resistance to TSWV and LS.

Genome sequence analysis of *A. duranensis* indicated presence of 652 genes in the above described region. Functional annotation of these genes demonstrated that 49 genes were defense related (Supplementary Table S4). Among them, there were two NB-ARC protein genes, three LRR receptor-like genes, three cytochrome P450 genes, four disease resistance-responsive (dirigent-like protein) genes, three WRKY family transcription factors, 15 pentatricopeptide (PPR) repeat protein genes, eight peroxidase genes, and 11 serine/threonine-protein phosphatase genes. The NBS-LRR family genes are known as the largest class of disease resistance (R) genes in plants (Meyers et al., 2003). The majority of R genes cloned by a map-based approach were NBS-LRR genes, for example, the rice blast disease resistance gene *Pi9, Pi36*, and *Pi5* (Qu et al., 2006; Liu et al., 2007; Lee et al., 2009), wheat leaf rust disease resistance gene *Lr10* and wheat powdery mildew resistance gene *Pm3* (Feuillet et al., 2003; Yahiaoui et al., 2004). WRKY transcription factors act as key regulators of many processes in plants. Previous studies showed that WRKY transcription factors also play important roles in pathogen defense (Rushton et al., 2010). Our earlier study reported the identification of WRKY genes in *A. duranensis* and *A. ipaensis*, and found the expression of some WRKY genes respond to SA and JA treatment. This result suggested that these WRKY transcription factors might play important roles in peanut defense reaction (Song et al., 2016). Serine/threonine kinases are also important factors in plant defense system. In tomato, *Pti1* encodes a serine/threonine kinase which was involved in the hypersensitive response (Zhou et al., 1995). In wheat, a serine/threonine kinase Stpk-V encoded by a powdery mildew resistance gene *Pm21*, conferred powdery mildew resistance in wheat (Cao et al., 2011). These results suggest that the NBS-LRR, WRKY and serine/threonine kinase genes in the genomic region could be the major genes for TWSV or LS resistance. To test this hypothesis, we need further fine mapping of these QTLs. The nearest markers for the defense related genes are listed in Supplementary Table S4.

### Application of SSR Markers in *Arachis* Wild Relatives

Many of the wild relatives of peanut exhibit high resistance to diseases, that could be used to improve cultivars. For example, many wild *Arachis* species including *A. duranensis* (PI 219823) and their interspecific derivatives were resistant or immune to rust under both field and laboratory conditions (Wynne et al., 1991). *A. duranensis* was also highly resistant to tikka leaf spot disease caused by *Cercospora arachidicola* (Seetharam et al., 1973; Pande and Rao, 2001). In order to use the resistance genes of wild peanuts, several approaches were developed, such as distant hybridization and development of synthetic amphidiploids. Recent studies showed that the development of synthetic amphidiploids could help to overcome the crossing barrier caused by ploidy difference between tetraploid cultivated peanut and diploid wild relatives. Several synthetic amphidiploids were developed, including the artificial tetraploid IpaDur (*A. ipaensis* × *A. duranensis* V14167) and ISATGR278-18 (*A. duranensis* × *A. batizocoi*) (Mallikarjuna et al., 2004, 2011; Kumari et al., 2014; Bertioli et al., 2016). After hybridization between cultivars and synthetic amphidiploids, molecular markers are needed for detection and tracking the DNA fragments from the wild type peanuts. The amphidiploid IpaDur was crossed with Fenghua 1, a high-yielding variety in China. Using one of the SSR markers (Araip.B07_130994) developed in this study, true F_1_ hybrids were easily identified (**Figure 5B**). In the future, we will create *A. hypogaea*–*A. duranensis* near-isogenic lines (NILs), which will be important materials for both peanut breeding and genetic studies. Using these SSR markers, the introgression of resistant genes and large DNA fragments from *A. duranensis* could be examined in the NILs.

In summary, we developed a large number of genomic SSR markers from diploid progenitors of cultivated peanut for deployment in an array of genetic and breeding applications. This study also demonstrated high transferability for these SSRs in different *Arachis* species, indicating high potential for their useful applications in improving cultivated peanut.

## Author Contributions

XW, BG, MKP, SW, and RKV conceived and designed the experiments. CZ, MKP, JQ, GA, JW, DB, and XR performed the experiments. CZ, MKP, HX, and CM analyzed data. CZ, XW, MKP, and BG wrote and revised the manuscript.

## Conflict of Interest Statement

The authors declare that the research was conducted in the absence of any commercial or financial relationships that could be construed as a potential conflict of interest.

## Footnotes

1 http://www.peanutsusa.com

2 http://www.tiangen.com/en/?productShow/t1/4/id/10.html

3 http://peanutbase.org/

4 http://pgrc.ipk-gatersleben.de/misa/

5 http://biotools.nubic.northwestern.edu/Primer3.html

6 http://www.ncbi.nlm.nih.gov/tools/epcr/

7 https://www.ncbi.nlm.nih.gov/genbank/

8 ftp://ftp.ncbi.nlm.nih.gov/blast/executables/blast+/LATEST/

## References

[B1] Balfourier F. Roussel V. Strelchenko P. Exbrayat-Vinson F. Sourdille P. Boutet G. (2007). A worldwide bread wheat core collection arrayed in a 384-well plate. *Theor. Appl. Genet.* 114 1265–1275. 17318494 10.1007/s00122-007-0517-1

[B2] Bertioli D. J. Cannon S. B. Froenicke L. Huang G. Farmer A. D. Cannon E. K. (2016). The genome sequences of *Arachis duranensis* and *Arachis ipaensis*, the diploid ancestors of cultivated peanut. *Nat. Genet.* 48 438–446. 10.1038/ng.3517 26901068

[B3] Cao A. Xing L. Wang X. Yang X. Wang W. Sun Y. (2011). Serine/threonine kinase gene Stpk-V, a key member of powdery mildew resistance gene *Pm21*, confers powdery mildew resistance in wheat. *Proc. Natl. Acad. Sci. U.S.A.* 108 7727–7732. 10.1073/pnas.1016981108 21508323 PMC3093467

[B4] Chen X. Li H. Pandey M. K. Yang Q. Wang X. Garg V. (2016). Draft genome of the peanut A-genome progenitor (*Arachis duranensis*) provides insights into geocarpy, oil biosynthesis, and allergens. *Proc. Natl. Acad. Sci. U.S.A.* 113 6785–6790. 10.1073/pnas.1600899113 27247390 PMC4914189

[B5] Cho Y. G. Ishii T. Temnykh S. Chen X. Lipovich L. McCouch S. R. (2000). Diversity of microsatellites derived from genomic libraries and GenBank sequences in rice (*Oryza sativa* L.). *Theor. Appl. Genet.* 100 713–722.

[B6] Deng P. Wang M. Feng K. Cui L. Tong W. Song W. (2016). Genome-wide characterization of microsatellites in Triticeae species: abundance, distribution and evolution. *Sci. Rep.* 6:32224 10.1038/srep32224 PMC4999822 27561724

[B7] Feuillet C. Travella S. Stein N. Albar L. Nublat A. Keller B. (2003). Map-based isolation of the leaf rust disease resistance gene *Lr10* from the hexaploid wheat (*Triticum aestivum* L.) genome. *Proc. Natl. Acad. Sci. U.S.A.* 100 15253–15258. 14645721 10.1073/pnas.2435133100 PMC299976

[B8] Guo B. Fedorova N. D. Chen X. Wan C. H. Wang W. Nierman W. C. (2011). Gene expression profiling and identification of resistance genes to *Aspergillus flavus* infection in peanut through EST and microarray strategies. *Toxins* 3 737–753. 10.3390/toxins3070737 22069737 PMC3202856

[B9] Gupta P. K. Balyan H. S. Sharma P. C. Ramesh B. (1996). Microsatellites in plants: a new class of molecular markers. *Curr. Sci.* 70 45–54.

[B10] Han B. Wang C. Tang Z. Ren Y. Li Y. Zhang D. (2015). Genome-wide analysis of microsatellite markers based on sequenced database in Chinese spring wheat (*Triticum aestivum* L.). *PLoS ONE* 10:e0141540 10.1371/journal.pone.0141540 PMC4633229 26536014

[B11] Haq S. U. Jain R. Sharma M. Kachhwaha S. Kothari S. L. (2014). Identification and characterization of microsatellites in expressed sequence tags and their cross transferability in different plants. *Int. J. Genomics* 2014:863948 10.1155/2014/863948 PMC4217358 25389527

[B12] Huang L. Wu B. Zhao J. Li H. Chen W. Zheng Y. (2016). Characterization and transferable utility of microsatellite markers in the wild and cultivated *Arachis* species. *PLoS ONE* 11:e0156633 10.1371/journal.pone.0156633 PMC4887017 27243460

[B13] Krishna G. Singh B. K. Kim E. K. Morya V. K. Ramteke P. W. (2015). Progress in genetic engineering of peanut (*Arachis hypogaea* L.)—A review. *Plant Biotechnol. J.* 13 147–162. 10.1111/pbi.12339 25626474

[B14] Kumari V. Gowda M. V. C. Tasiwal V. Pandey M. K. Bhat R. S. Mallikarjuna N. (2014). Diversification of primary gene pool through introgression of resistance to foliar diseases from synthetic amphidiploids to cultivated groundnut (*Arachis hypogaea* L.). *Crop J.* 2 110–119.

[B15] Lavia G. I. Ortiz A. M. Robledo G. Fernández A. Seijo G. (2011). Origin of triploid *Arachis pintoi* (Leguminosae) by autopolyploidy evidenced by FISH and meiotic behaviour. *Ann. Bot.* 108 103–111. 10.1093/aob/mcr108 21693666 PMC3119619

[B16] Lee S. K. Song M. Y. Seo Y. S. Kim H. K. Ko S. Cao P. J. (2009). Rice Pi5-mediated resistance to *Magnaporthe oryzae* requires the presence of two coiled-coil–nucleotide-binding–leucine-rich repeat genes. *Genetics* 181 1627–1638. 10.1534/genetics.108.099226 19153255 PMC2666525

[B17] Liang X. Chen X. Hong Y. Liu H. Zhou G. Li S. (2009). Utility of EST-derived SSR in cultivated peanut (*Arachis hypogaea* L.) and *Arachis* wild species. *BMC Plant Biol.* 9:35 10.1186/1471-2229-9-35 PMC2678122 19309524

[B18] Liu F. Hu Z. Liu W. Li J. Wang W. Liang Z. (2016). Distribution, function and evolution characterization of microsatellite in *Sargassum thunbergii* (Fucales, Phaeophyta) transcriptome and their application in marker development. *Sci. Rep.* 6:18947 10.1038/srep18947 PMC4702172 26732855

[B19] Liu X. Lin F. Wang L. Pan Q. (2007). The *in silico* map-based cloning of *Pi36*, a rice coiled-coil–nucleotide-binding site–leucine-rich repeat gene that confers race-specific resistance to the blast fungus. *Genetics* 176 2541–2549. 17507669 10.1534/genetics.107.075465 PMC1950653

[B20] Mallikarjuna N. Pande S. Jadhav D. R. Sastri D. C. Rao J. N. (2004). Introgression of disease resistance genes from *Arachis kempff-mercadoi* into cultivated groundnut. *Plant Breed.* 123 573–576.

[B21] Mallikarjuna N. Senthilvel S. Hoisington D. (2011). Development of new sources of tetraploid *Arachis* to broaden the genetic base of cultivated groundnut (*Arachis hypogaea* L.). *Genet. Resour. Crop Evol.* 58 889–907.

[B22] Meyers B. C. Kozik A. Griego A. Kuang H. Michelmore R. W. (2003). Genome-wide analysis of NBS-LRR–encoding genes in Arabidopsis. *Plant Cell* 15 809–834. 12671079 10.1105/tpc.009308 PMC152331

[B23] Pande S. Rao J. N. (2001). Resistance of wild *Arachis* species to late leaf spot and rust in greenhouse trials. *Plant Dis.* 85 851–855. 10.1094/PDIS.2001.85.8.851 30823052

[B24] Pandey M. K. Agarwal G. Kale S. M. Clevenger J. Nayak S. N. Sriswathi M. (2017). Development and evaluation of a high density genotyping ‘Axiom_*Arachis*’ array with 58K SNPs for accelerating genetics and breeding in groundnut. *Sci. Rep.* 7:40577 10.1038/srep40577 PMC5238394 28091575

[B25] Pandey M. K. Monyo E. Ozias-Akins P. Liang X. Guimarães P. Nigam S. N. (2012). Advances in *Arachis* genomics for peanut improvement. *Biotechnol. Adv.* 30 639–651. 10.1016/j.biotechadv.2011.11.001 22094114

[B26] Pandey M. K. Roorkiwal M. Singh V. K. Ramalingam A. Kudapa H. Thudi M. (2016). Emerging genomic tools for legume breeding: current status and future prospects. *Front. Plant Sci.* 7:455 10.3389/fpls.2016.00455 PMC4852475 27199998

[B27] Powell W. Machray G. C. Provan J. (1996). Polymorphism revealed by simple sequence repeats. *Trends Plant Sci.* 1 215–222.

[B28] Qu S. Liu G. Zhou B. Bellizzi M. Zeng L. Dai L. (2006). The broad-spectrum blast resistance gene Pi9 encodes a nucleotide-binding site–leucine-rich repeat protein and is a member of a multigene family in rice. *Genetics* 172 1901–1914. 16387888 10.1534/genetics.105.044891 PMC1456263

[B29] Rushton P. J. Somssich I. E. Ringler P. Shen Q. J. (2010). WRKY transcription factors. *Trends Plant Sci.* 15 247–258. 10.1016/j.tplants.2010.02.006 20304701

[B30] Seetharam A. Nayar K. M. D. Sreekantaradhya R. Achar D. K. T. (1973). Cytological studies on the interspecific hybrid of *Arachis hypogaea* × *Arachis duranensis*. *Cytologia* 38 277–280.

[B31] Shi J. Huang S. Zhan J. Yu J. Wang X. Hua W. (2014). Genome-wide microsatellite characterization and marker development in the sequenced Brassica crop species. *DNA Res.* 21 53–68. 10.1093/dnares/dst040 24130371 PMC3925394

[B32] Shirasawa K. Bertioli D. J. Varshney R. K. Moretzsohn M. C. Leal-Bertioli S. C. Thudi M. (2013). Integrated consensus map of cultivated peanut and wild relatives reveals structures of the A and B genomes of *Arachis* and divergence of the legume genomes. *DNA Res.* 20 173–184. 10.1093/dnares/dss042 23315685 PMC3628447

[B33] Shore J. Parida S. K. Yadava D. K. Mohapatra T. (2010). Microsatellites in *Brassica* unigenes: relative abundance, marker design, and use in comparative physical mapping and genome analysis. *Genome* 53 55–67. 10.1139/g09-084 20130749

[B34] Song G. Q. Li M. J. Xiao H. Wang X. J. Tang R. H. Xia H. (2010). EST sequencing and SSR marker development from cultivated peanut (*Arachis hypogaea* L.). *Electr. J. Biotechnol.* 13 7–8.

[B35] Song H. Wang P. Lin J. Y. Zhao C. Bi Y. Wang X. (2016). Genome-wide identification and characterization of WRKY gene family in peanut. *Front. Plant Sci.* 7:534 10.3389/fpls.2016.00534 PMC4845656 27200012

[B36] Squirrell J. Hollingsworth P. M. Woodhead M. Russell J. Lowe A. J. Gibby M. (2003). How much effort is required to isolate nuclear microsatellites from plants? *Mol. Ecol.* 12 1339–1348. 12755865 10.1046/j.1365-294x.2003.01825.x

[B37] Varshney R. K. Mohan S. M. Gaur P. M. Gangarao N. V. P. R. Pandey M. K. Bohra A. (2013). Achievements and prospects of genomics-assisted breeding in three legume crops of the semi-arid tropics. *Biotechnol. Adv.* 31 1120–1134. 10.1016/j.biotechadv.2013.01.001 23313999

[B38] Varshney R. K. Thiel T. Stein N. Langridge P. Graner A. (2002). *In silico* analysis on frequency and distribution of microsatellites in ESTs of some cereal species. *Cell. Mol. Biol. Lett.* 7 537–546. 12378259

[B39] Wang H. Pandey M. K. Qiao L. Qin H. Culbreath A. K. He G. (2013). Genetic mapping and quantitative trait loci analysis for disease resistance using F2 and F5 generation-based genetic maps derived from ‘Tifrunner’ × ‘GT-C20’in peanut. *Plant Genome* 6 1–10.

[B40] Wang H. Penmetsa R. V. Yuan M. Gong L. Zhao Y. Guo B. (2012). Development and characterization of BAC-end sequence derived SSRs, and their incorporation into a new higher density genetic map for cultivated peanut (*Arachis hypogaea* L.). *BMC Plant Biol.* 12:10 10.1186/1471-2229-12-10 PMC3298471 22260238

[B41] Wang Q. Fang L. Chen J. Hu Y. Si Z. Wang S. (2015). Genome-wide mining, characterization, and development of microsatellite markers in *Gossypium* species. *Sci. Rep.* 5:10638 10.1038/srep10638 PMC4650602 26030481

[B42] Wynne J. C. Beute M. K. Nigam S. N. (1991). Breeding for disease resistance in peanut (*Arachis hypogaea* L.). *Annu. Rev. Phytopathol.* 29 279–303.

[B43] Yahiaoui N. Srichumpa P. Dudler R. Keller B. (2004). Genome analysis at different ploidy levels allows cloning of the powdery mildew resistance gene *Pm3b* from hexaploid wheat. *Plant J.* 37 528–538. 14756761 10.1046/j.1365-313x.2003.01977.x

[B44] Zhang J. Liang S. Duan J. Wang J. Chen S. Cheng Z. (2012). De novo assembly and characterization of the transcriptome during seed development, and generation of genic-SSR markers in Peanut (*Arachis hypogaea* L.). *BMC Genomics* 13:90 10.1186/1471-2164-13-90 PMC3350410 22409576

[B45] Zhong R. Zhou M. Zhao C. Hou L. Li C. Wang X. (2016). SSR marker development from peanut gynophore transcriptome sequencing. *Plant Breed.* 135 111–117.

[B46] Zhou J. Loh Y. T. Bressan R. A. Martin G. B. (1995). The tomato gene pti1 encodes a serine/threonine kinase that is phosphorylated by *Pto* and is involved in the hypersensitive response. *Cell* 83 925–935. 8521516 10.1016/0092-8674(95)90208-2

[B47] Zhou X. Dong Y. Zhao J. Huang L. Ren X. Chen Y. (2016). Genomic survey sequencing for development and validation of single-locus SSR markers in peanut (*Arachis hypogaea* L.). *BMC Genomics* 17:420 10.1186/s12864-016-2743-x PMC4888616 27251557

